# Vascular Sema3E-Plexin-D1 Signaling Reactivation Promotes Post-stroke Recovery through VEGF Downregulation in Mice

**DOI:** 10.1007/s12975-021-00914-4

**Published:** 2021-05-12

**Authors:** Ri Yu, Nam-Suk Kim, Yan Li, Jin-Young Jeong, Sang-Joon Park, Bin Zhou, Won-Jong Oh

**Affiliations:** 1 Neurovascular Biology Laboratory, Neurovascular Unit Research Group, Korea Brain Research Institute, Daegu, 41062 Republic of Korea; 2 College of Veterinary Medicine, Kyungpook National University, Daegu, 41566 Republic of Korea; 3 Department of Brain and Cognitive Sciences, Daegu Gyeongbuk Institute of Science and Technology, Daegu, 42988 Republic of Korea; 4 Institute of Biochemistry and Cell Biology, Shanghai Institutes for Biological Sciences, Chinese Academy of Sciences, Shanghai, 200031 China

**Keywords:** Plexin-D1, Axon guidance, Stroke, Vascular recovery, Blood–brain barrier

## Abstract

Post-stroke vascular remodeling, including angiogenesis, facilitates functional recovery. Proper vascular repair is important for efficient post-stroke recovery; however, the underlying mechanisms coordinating the diverse signaling pathways involved in vascular remodeling remain largely unknown. Recently, axon guidance molecules were revealed as key players in injured vessel remodeling. One such molecule, Semaphorin 3E (Sema3E), and its receptor, Plexin-D1, control vascular development by regulating vascular endothelial growth factor (VEGF) signaling. In this study, using a mouse model of transient brain infarction, we aimed to investigate whether Sema3E-Plexin-D1 signaling was involved in cerebrovascular remodeling after ischemic injury. We found that ischemic damage rapidly induced *Sema3e* expression in the neurons of peri-infarct regions, followed by Plexin-D1 upregulation in remodeling vessels. Interestingly, Plexin-D1 reemergence was concurrent with brain vessels entering an active angiogenic process. In line with this, *Plxnd1* ablation worsened neurological deficits, infarct volume, neuronal survival rate, and blood flow recovery. Furthermore, reduced and abnormal vascular morphogenesis was caused by aberrantly increased VEGF signaling. In *Plxnd1* knockout mice, we observed significant extravasation of intravenously administered tracers in the brain parenchyma, junctional protein downregulation, and mislocalization in regenerating vessels. This suggested that the absence of Sema3E-Plexin-D1 signaling is associated with blood–brain barrier (BBB) impairment. Finally, the abnormal behavioral performance, aberrant vascular phenotype, and BBB breakdown defects in *Plxnd1* knockout mice were restored following the inhibition of VEGF signaling during vascular remodeling. These findings demonstrate that Sema3E-Plexin-D1 signaling can promote functional recovery by downregulating VEGF signaling in the injured adult brain.

**Supplementary Information:**

The online version contains supplementary material available at 10.1007/s12975-021-00914-4.

## Introduction

Stroke is the most common cause of death and a leading cause of disability worldwide [1, 2]. It is accompanied by structural and functional vascular injuries and remodeling [3]; therefore, enhancing angiogenesis in the damaged tissue to promote recovery of nearby brain cells has been proposed as a therapeutic option [3, 4]. However, despite tremendous efforts to develop pharmacological therapies promoting cerebrovascular repair, there are no definitive remedies. This is mainly due to the varying degrees of severity from early-onset to late chronic pathology and the multifaceted cellular and molecular events that occur during repair [5–7]. For example, treatment with tissue plasminogen activator is considered the gold standard for ischemic stroke; however, one of its side effects is an enhanced inflammatory response in brain capillaries and neuronal cell damage [8, 9]. The brain vasculature has a unique structure that is composed of endothelial cells, neurons, astrocytes, and pericytes. This is known as the neurovascular unit (NVU), where vessels tightly regulate the passage of substances between the bloodstream and brain parenchyma via the blood–brain barrier (BBB) [10, 11]. Neural activity and vascular dynamics are tightly coupled in the NVU; the connection and bidirectional communication between the two systems is crucial for proper brain function [12, 13]. Because of the importance of such integrative interaction, neovascularization and vascular repair in the brain after injury require stepwise processes and regulatory mechanisms to reconstruct a functional NVU [14–16].

Previous studies have shown that angiogenesis rapidly occurs post-ischemia in the brain. This contributes to functional recovery and prolonged survival in human patients and experimental rodent models [3, 16, 17]. Indeed, endothelial cell proliferation occurs at 12–24 h following a stroke, and active angiogenesis in the peri-infarcted region is observed 3–4 days after an ischemic insult in humans [15]. However, little is known about the molecular mechanism orchestrating vascular remodeling during repair processes in the post-ischemic brain. To coordinate a highly branched and hierarchically organized vascular network, endothelial cells grow and connect according to the multiple steps during angiogenesis [18]. VEGF and its receptor, VEGF receptor 2 (VEGFR2), are expressed by endothelial cells; they are master mediators of vascular network establishment during both developmental and pathological angiogenesis [19, 20]. After cerebral ischemia, both VEGF and VEGFR2 are mainly expressed in the ischemic border, known as the peri-infarct region, where the vessels remain salvageable. Therefore, this is considered a treatable target [21–23]. Interestingly, VEGF signaling is associated with neuroprotection and neurogenesis in ischemic stroke models [24–28]. However, it produces harmful effects, such as BBB breakdown, that are dependent on administration timing and dosage after stroke induction [29, 30]. Therefore, elucidating the balanced mechanism and precise activation dynamics of VEGF signaling is of high importance [31].

During the angiogenic process, endothelial tip cells use the same guidance molecules as neuronal growth cones to regulate proper vascular network formation [10, 12]. These cues also play a crucial role during vascular remodeling induced by ischemic damage [32]. One such guidance cue is Semaphorin 3E (Sema3E) and its receptor, Plexin-D1. Sema3E is one of seven class-3 secreted semaphorins. It transduces its signal via direct binding to the Plexin-D1 receptor independently of neuropilins, the primary binding receptor of other class-3 semaphorins [13, 33]. In the nervous system, the Sema3E-Plexin-D1 axis provides a repulsive signal by inhibiting axonal growth or restricting synapse formation [34, 35]. However, its mode of action on vascular topology varies depending on the temporal and spatial expression patterns of Sema3E and Plexin-D1 [33]. For example, the absence of Sema3E-Plexin-D1 signaling leads to excessive vessel outgrowth in the developing somite or decreased vessel sprouting in the retina [13, 36]. Moreover, Sema3E-Plexin-D1 signaling is associated with ischemia-induced angiogenesis in the periphery tissue and retina [13, 37], but its activity in the central nervous system has not been elucidated. A few studies have revealed that Sema3E-Plexin-D1 signaling is interwoven with VEGF signaling under normal or pathological conditions [36–38]. In the developing and pathological retinal vasculature, Sema3E-Plexin-D1 signaling modulates the angiogenic process at sprouting ends through a VEGF-induced negative feedback mechanism [36, 37]. Because Sema3E-Plexin-D1 signaling is actively involved in many angiogenic conditions, we speculated that this signaling contributes to vascular remodeling after brain damage, such as in ischemic stroke. In this study, we investigated whether Sema3E-Plexin-D1 signaling was necessary for the development of functional brain vasculature during vascular remodeling after an ischemic insult.

## Materials and Methods

### Animals

*Plxnd1*^*flox/flox*^ [36] and *Mfsd2a-CreERT2* mice [39] were maintained on a C57Bl/6 J (#000664, Jax) background. *Rosa26-RFP* mice (( Ai9; #007909, Jax)) were purchased from the Jackson Laboratory and maintained on a C57Bl/6 J background. To generate the endothelial cell-specific inducible *Plxnd1* knockout mice, tamoxifen (T5648, Sigma) was dissolved in corn oil and administered to male mice 14 days before surgery. Male mice received 0.1–0.2 mg tamoxifen per gram of body weight by oral gavage. All protocols for animal experiments were approved by the Institutional Animal Care and Use Committee of Korea Brain Research Institute (IACUC-18–00008). All experiments were performed according to the National Institutes of Health Guide for the Care and Use of Laboratory Animals and ARRIVE guidelines.

### Ischemic Brain Mouse Model

The transient middle cerebral artery occlusion (tMCAO) mouse model, whereby a temporary reduction of blood flow followed by reperfusion generates cerebral ischemic injury in the cortex and striatum, was performed as described previously [40]. Briefly, anesthesia was induced with 5% isoflurane in an anesthetic chamber and maintained with 1.5% isoflurane via a nose cone. Rectal temperature was maintained at 37.0 ± 0.5 °C during surgery and the recovery period using a 37 °C heating pad. A 1-cm-long midline skin incision was performed in the neck area. The right common carotid artery (CCA) was carefully dissected free from the surrounding nerves and tied off using a 7–0 silk suture. The right external carotid artery was isolated and cauterized 2–3 mm distal to the bifurcation, and a vessel clip was placed just before the CCA bifurcation to avoid retrograde flow at the time of arteriotomy. An arteriotomy was performed between the proximal ligated suture and vessel clip on the CCA with micro-scissors. Next, a silicone-rubber-coated 6–0 nylon filament (602145PK10, Doccol) was inserted into the CCA and advanced for 9 mm to the carotid bifurcation along the internal carotid artery (ICA) and origin of the middle cerebral artery (MCA). Next, the neck incision was sutured and the mouse was placed in a 37 °C nursing box to recover from anesthesia. After 30 min of occlusion, the filament was removed to restore the blood flow to the MCA. In sham-operated mice, the ICA was surgically prepared for filament insertion, but it was not inserted.

### In Situ* Hybridization*

In situ hybridization (ISH) was performed under RNase-free conditions by standard procedures [36]. After fixation in 4% paraformaldehyde (PFA) for 20 min, 14-μm-thick cryosections sections were preincubated in hybridization buffer (5 × Denhardt’s solution, 5 × saline sodium citrate (SSC), 50% formamide, 0.25 mg/ml Baker yeast tRNA, and 0.2 mg/ml salmon sperm DNA) for at least 1 h at room temperature. Next, sections were hybridized in the same buffer containing the indicated digoxigenin-conjugated riboprobe at 60 °C overnight. After hybridization, sections were washed in serial SSC buffer, formamide solution, and preincubated in buffer 1 (100 mM Tris–HCl, pH 7.5, 150 mM NaCl) plus 1% blocking reagent (Roche) for 1 h at room temperature. Next, sections were incubated with sheep anti-digoxigenin-alkaline phosphatase (AP) antibody (1:3000, Roche) for 90 min at room temperature, washed in the buffer 1, and incubated in AP buffer (100 mM Tris–HCl, pH 9.5, 100 mM NaCl, 5 mM MgCl_2_) containing 4-nitro blue tetrazolium chloride (NBT, Roche), 5-bromo-4-chloro-3-indolyl-phosphate (BCIP, Roche), and levamisole (1359302, Sigma). Purple precipitates were visualized. After mounting with coverslips, the samples were analyzed via confocal laser-scanning microscopy with a Nikon Eclipse Ti-U microscope or Leica TCS SP8 confocal microscope.

For subsequent immunostaining with anti-ColIV (1:500, ab6586, Abcam) after ISH, sections were washed in 1 × phosphate-buffered saline (PBS) several times and post-fixed in 4% PFA for 5 min. After fixation, all procedures were performed as described in the immunohistochemistry section. For the double fluorescence ISH, the tyramide signal amplification method with minor modification was used according to the manufacturer’s instructions (NEL753001KT, PerkinElmer) [34]. The following anti-sense riboprobes were used: *Plxnd1*[36], *Sema3e*[13], and *Snap25* riboprobes were designed based on the ISH database provided by the Allen Institute for Brain Science.

### AP-Ligand Preparation and Binding Analysis

AP-conjugated Sema3E ligands were generated in HEK293T cells, and ligand binding experiments were performed as described previously [36]. Briefly, the AP-Sema3E expression construct was transfected into cells by LipofectAMINE 2000 (11668019, Invitrogen) and cultured overnight in Dulbecco’s Modified Eagle’s Medium (DMEM, 11995–065, Gibco) containing 10% fetal bovine serum (FBS). Next, the medium was replaced with OPTI-MEM (31985–070, Gibco) and harvested at 5 days post-transfection. The collected conditioned medium was filtered to increase ligand concentration.

For AP-Sema3E binding analysis, 20-μm-thick cryosections were fixed in cold methanol for 8 min and preincubated in 1 × PBS containing 4 mM MgCl_2_ and 10% FBS for 1 h. Next, a binding solution (1 × PBS-MgCl_2_ plus 20 mM HEPES, pH 7.0) containing 2 nM AP-Sema3E was applied, and sections were incubated for 2 h at room temperature. After five washes in 1 × PBS-MgCl_2_, sections were briefly soaked in acetone-formaldehyde fixative (60% acetone, 1.1% formaldehyde, and 20 mM HEPES pH 7.0) and heat-inactivated in 1 × PBS at 65 °C for 2 h. Next, sections were incubated in AP buffer with NBT and BCIP overnight at room temperature. The subsequent immunostaining procedure was performed as described in the immunohistochemistry section. For quantification, three brain sections per animal were analyzed and averaged the mean signal intensity per image.

### Neurological Scoring

A neurological deficit grading system [41] was used to evaluate mice at 1, 2, 3, 4, 5, 6, and 7 days after tMCAO. The scores were as follows: 0, no neurological deficit; 1, forelimb flexion when suspended by the tail, or failure to fully extend forepaw; 2, shoulder adduction when suspended by the tail; 3, reduced resistance to lateral push; 4, spontaneous movement in all directions with unilateral circling only if pulled by the tail; 5, spontaneous unilateral circling; 6, walk only when stimulated; 7, no response to stimulation; and 8, stroke-related death.

### Magnetic Resonance Imaging

Magnetic resonance imaging (MRI) experiments were performed using a Bruker 9.4 preclinical MRI (BioSpec 94/20 USR; Laboratory Animal Center of Daegu-Gyeongbuk Medical Innovation Foundation, Korea). Briefly, mice were anesthetized with a continuous supply of 1.5% isoflurane. Respiratory rates were monitored continuously. A fast spin-echo sequence (4000/12.5 ms of repetition time/echo time) was employed for T2-weighted imaging and a spin-echo sequence (700/7 ms of repetition time/echo time) for T1-weighted imaging. For all scans, the field of view was 20 × 20 mm^2^, slice thickness 3.0 mm, number of slices 18, and matrix size 256 × 256, except for the diffusion sequence where the matrix size was 128 × 128. Pre-contrast T1-weighted images were acquired before gadoterate meglumine (Gd-DOTA) injection. Post-contrast T1-weighted images were acquired 30 min after intravenous injection of Gd-DOTA (0.2 ml/kg body weight) with normal saline. A region of interest (ROI) was drawn in the peri-infarct and extravasation areas on images using the ImageJ software (National Institutes of Health, Bethesda, MD; http://imagej.nih.gov/ij/).

### Immunohistochemistry

For immunostaining with floating samples, brains were fixed in 4% PFA overnight and equilibrated with 30% sucrose in 1 × PBS. Mouse brain sections were cut into 30-μm slices on a cryostat (Leica Microsystems Inc., Buffalo Grove, IL, USA). Next, they were permeabilized in PBST (PBS containing 0.3% Triton X-100) for 10 min, blocked with 1% bovine serum albumin (BSA) in PBST for 60 min at room temperature, and incubated in primary antibody diluted in blocking solution overnight at 4 °C. The following primary antibodies were used: anti-CD31 (1:500, 553370, BD Bioscience), anti-Collagen IV (1:500, ab6586, Abcam), anti-NeuN (1:1000, MAB377, Merck), anti-Ter-119 (1:100, MAB1125, R&D System), and anti-MAP-2 (1:500, ab32454, Abcam). After washing with PBST three times, sections were incubated for 1 h with Alexa Fluor 488-, 594-, or 647-conjugated secondary antibodies (1:1000, Invitrogen). For negative controls, brain sections were stained with secondary antibodies alone.

For BBB junctional protein immunostaining, brains were isolated, immediately snap-frozen with liquid nitrogen, and cut (14-μm-thick sections) on a cryostat. Sections were fixed in cold 100% acetone for 15 min followed by cold 100% methanol for 20 min. Permeabilization, blocking, and antibody incubation were performed as described above. The following primary antibodies were used: anti-ZO-1 (1:500, 61–7300, ThermoFisher), anti-claudin5 (1:200, 35–2500, Invitrogen), anti-claudin5-488 ( 1:500, 352588, Invitrogen), and anti-Occludin (1:200, 33–1500, Invitrogen). Images were collected under a Nikon Eclipse Ti-U microscope (Nikon, Japan) or Leica TCS SP8 confocal microscope (Leica, Germany). Image processing was performed using the ImageJ, Adobe Photoshop (Adobe Photoshop CC2019), and Angio Tool (National Cancer Institute, Gaithersburg, MD, USA) software.

### Measurements of Infarct Volume

Infarct area was measured by cresyl violet and microtubule-associated protein 2 (MAP2) staining. Mouse brain sections were cut into 30-μm slices on a cryostat (Leica Microsystems Inc., Buffalo Grove, IL, USA) from five levels (bregma −1.6 mm, −0.8 mm, 0 mm, 0.8 mm, and 1.6 mm) and stained with 1% cresyl violet solution. In addition, the neuron-specific marker, MAP2, was visualized with anti-MAP2 (1:500, ab32454, Abcam) immunofluorescence staining. Sections were digitized and the border between infarct and non-infarct tissue outlined using ImageJ. The infarct volume was calculated by subtracting the volume of the non-lesioned area in the ipsilateral hemisphere from the volume of the whole area in the contralateral hemisphere.

### Vascular Labeling and Analysis of Vascular Density

Animals were intravenously perfused 5 min before euthanasia with Dylight 594-conjugated *Lycopersicon esculentum (Tomato) lectin* (DL-1177, Vector Laboratories) at a dose of 1 mg/kg body weight. After perfusion, the brains were dissected, fixed, and then cut into 100-μm-thick free-floating sections using a vibratome. Vascular area (%), vessel length (μm/μm^2^), and branch point number were calculated using Angio Tool.

For microaneurysm-like capillary vessel analysis, images immunostained with anti-Col IV and anti-Ter-119 antibodies without PBS perfusion procedure were analyzed using ImageJ. The capillaries holding the single lining of red blood cells were defined as a normal capillary. The mean diameter of the capillaries was calculated to determine the cutoff value. Vessels thicker than the cutoff size were considered abnormal. The number and coverage of abnormal *vs* normal vessels were analyzed per image.

### Terminal Deoxynucleotidyl Transferase–Mediated dUTP Nick End-Labeling Staining

Terminal deoxynucleotidyl transferase dUTP nick end labeling (TUNEL) assay was employed to observe the apoptotic cells. Using the In Situ Cell Death Detection Kit, TMR red (12156792910, Roche), the brain sections were treated according to the manufacturer’s protocol.

### Immunoblotting

Brain tissue was collected in a RIPA buffer with a protease inhibitor cocktail (11697498001, Roche) and protein amounts were quantified by a BCA protein assay kit (23227, Thermo Fisher Scientific). A total of 40 g of protein was loaded per well and separated on a sodium dodecyl sulfate (SDS) polyacrylamide gel and transferred to a polyvinylidene fluoride (PVDF) membrane (IPVH00010, Merck) at 100 V for 90 min. All membranes were blocked with Everyblot blocking buffer (12010020, Bio-rad) for 1 h and probed overnight with the indicated primary antibodies in blocking buffer at 4 °C. The primary antibodies were rabbit monoclonal anti-Phospho-VEGF Receptor2 (Tyr951) (15D2) antibody (1:1000, 4991, Cell Signaling), rabbit monoclonal anti-Phospho-VEGF Receptor 2 (Tyr1175) (D5B11) antibody (1:1000, 3770, Cell Signaling), rabbit monoclonal anti-VEGF Receptor 2 (55B11) antibody (1:1000, 2479, Cell Signaling), goat polyclonal anti-Dll4 antibody (1:1000, AF1389, R&D systems), rabbit monoclonal anti-b-actin antibody (1:5000, 5125, Cell Signaling), rabbit polyclonal anti-ZO-1 antibody (1:1000, 61–7900, Invitrogen), rabbit polyclonal anti-Claudin-5 antibody (1:1000, 34–1600, Invitrogen), and rabbit polyclonal anti-Occludin antibody (1:1000, 71–1500, Invitrogen). The membranes were incubated in PBST and appropriate horseradish peroxidase (HRP)–conjugated secondary antibodies. Bands were developed with enhanced chemiluminescence using the Fusion FX7 (Vilber, Germany) and analyzed using Image J.

### Whole-Brain Immunostaining and Clearing

For adult mouse brain clearing, the CUBIC method was used [42] according to the manufacturer’s instructions (T3740 and T3741, Tokyo Chemical Industry). After fixation in cold 4% PFA in 0.1 M phosphate buffer (pH 7.4), the areas of interest in the brain were sectioned in a parallel direction (3 mm) and washed with 1 × PBS three times for at least 2 h each time with shaking at room temperature (RT). The samples were then cleared with 1/2-CUBIC-L under shaking at 60 rpm and 37 °C for 5 h. CUBIC-L was applied for clearing under the same conditions for 48 h. The samples were washed with 1 × PBS containing 0.3% Triton X-100 (0.3% PBST) with soft shaking at RT three times for at least 2 h each time, once overnight and again for 2 h. Next, samples were blocked with 5% BSA with shaking at 60 rpm at 37 °C for 3 h, followed by incubation with CD31 antibodies (diluted in 5% BSA) with shaking at 60 rpm at 37 °C for 24 h, and washing three times with PBST and shaking at RT for at least 1 h each time. Corresponding secondary antibodies (diluted in 5% BSA) were applied to the sample under the same conditions as primary antibody incubations. Next, the samples were rewashed with PBST three times for at least 1 h. Finally, 1/2-CUBIC-R was applied to clear samples with shaking at 60 rpm at 37 °C for 24 h. CUBIC-R was used for further clearing; it was replaced every 2 days until satisfactory optical transparency was achieved. Cleared samples were stored in CUBIC-R at 4 °C until imaging. Light-sheet microscopy images were acquired with the LightsheetZ.1 and 5 × EC plan objective lens (Carl Zeiss, Germany).

### Cortical Cerebral Blood Flow Measurement

Real-time two-dimensional cerebral blood flow (CBF) was monitored using a laser speckle contrast imager (PeriCam PSI HR System, Perimed, Sweden). After anesthesia induction, mice were placed in the prone position and the skull was exposed through a cut in the skin at the parietal midline. A camera was placed above the head at a working distance of 10 cm from the skull surface. This was illuminated with a laser diode (785 nm) to allow laser penetration through the brain. CBF was recorded before the induction of cerebral ischemia, during cerebral ischemia, and for the first 10 min of reperfusion. To evaluate CBF changes, the ROI included the cortical area supplied by the MCA.

### BBB Permeability

To assess BBB permeability, Texas red dextran (MW = 10 kDa, Invitrogen; 0.1 ml of 10 mg/ml) was injected retro-orbitally on 1, 3, and 7 days post-tMCAO. After a 2-h circulation period, mice were perfused transcardially with PBS, followed by ice-cold 4% PFA in 0.1 M phosphate buffer, and brains were dissected. Brain samples were post-fixed in 4% PFA for 16–18 h and equilibrated with 30% sucrose in 1 × PBS. Mouse brain sections that encompassed the MCA region were cut into 30-μm slices. Immunofluorescence was performed with antibodies for anti-CD31 (1:500, 553370, BD Bioscience). Alexa594-conjugated mouse IgG (1:500) was used to visualize IgG leakage in the brain. Sections were imaged with a Leica TCS SP8 confocal microscope (Leica, Germany). IgG leakage was quantified with the Fiji software. Brain slices were uniformly thresholded to quantify the total IgG area. Areas that exceeded threshold levels were defined as leakage areas. The histological procedure was performed as described in the immunohistochemistry section above.

### VEGF-R2 Inhibition

According to the manufacturer’s recommendations, mice received daily intraperitoneal injections (50 mg/kg) of VEGF-R2 inhibitor SU5416 (S8442, Sigma) dissolved in dimethylformamide (DMF). Control animals received intraperitoneal injections of DMF alone.

### Statistical Analysis

Statistical analyses were performed using Prism 8 (GraphPad Software). All results are presented as mean ± standard error of mean (SEM). At least three mice pairs per experiment were used for all histological analyses. For image data quantification, at least three brain sections per mouse were collected and analyzed. All data analyses were performed by a blinded investigator. Statistical significance was determined using two-tailed Mann–Whitney *U* tests between two groups and one- or two-way ANOVA with Tukey’s multiple comparisons test for three or more groups. For ANOVA analysis, the normal distribution of data was using the Shapiro–Wilk normality and Kolmogorov–Smirnov tests. Differences were considered as statistically significant when *p* < 0.05.

## Results

### Sema3E Is Rapidly Expressed in Peri-infarct Neurons, whereas Plexin-D1 Expression Slowly Increases in Blood Vessels

**Fig. 1:**
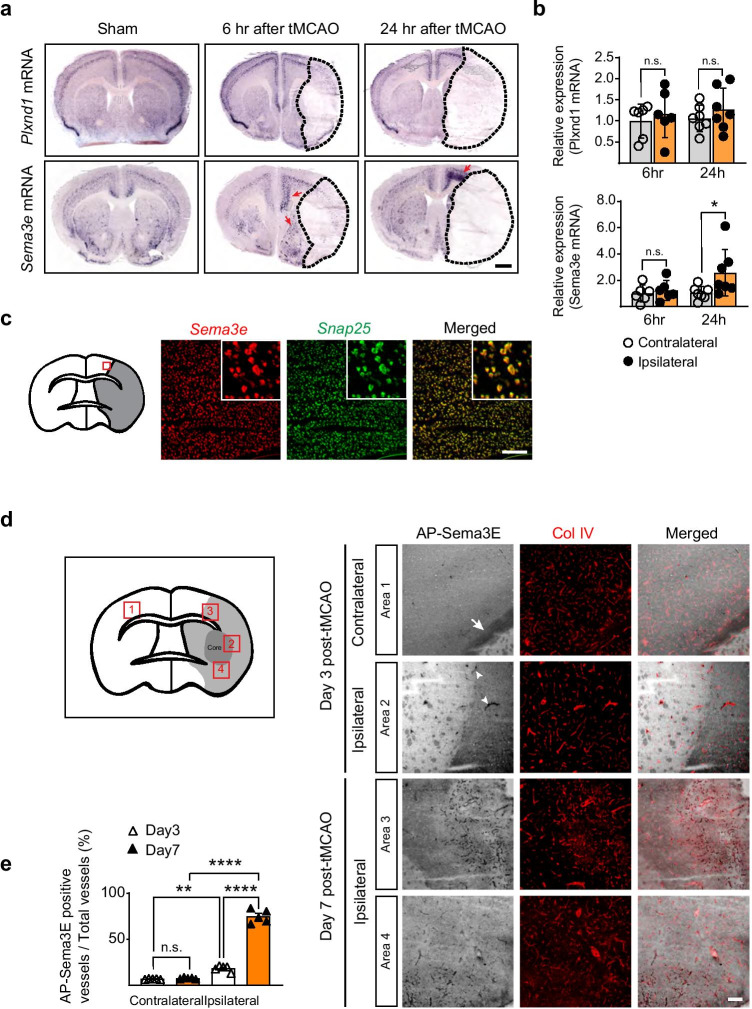
*Sema3e* is rapidly expressed in neurons in the peri-infarct region, whereas *Plxnd1* is induced in the remodeling vasculature **a** In situ hybridization of *Sema3e* and *Plxnd1* at 6 and 24 h after tMCAo. The expression of *Sema3e* mRNA (red arrows), but not *Plxnd1* mRNA, was increased in the peri-infarct region. Dotted lines mark regions damaged by tMCAo. Scale bar = 1 mm. **b** Real-time qPCR for quantitative analysis of mRNA expression in the peri-infarct tissue at 6 and 24 h after tMCAo (*n* = 6 mice/6 h group, *n* = 7 mice/24 h group). Data are shown as mean ± SEM. *p < 0.05; two-tailed Student’s *t*-test.** c** Double fluorescence ISH shows colocalization of *Sema3e* (red) and *snap25* (green) mRNA in the tissue adjacent to the ischemic region at 24 h after tMCAo. The red box indicates the analyzed brain area, insets are high-resolution images. Scale bar = 100 μm. **d** AP-Sema3E binding assay to detect Plexin-D1 expression in brain sections (black, left panels) on days 3 and 7 post-tMCAo, and vessels visualized by subsequent collagen IV (Col IV) immunostaining to detect blood vessels (red, middle panels). Three days after ischemic damage, Plexin-D1-positive vessels (white arrowheads) began to appear on the ipsilateral side, whereas no Plexin-D1 expression was observed on vessels other than those of the neuronal tracts (white arrow) on the contralateral side. Seven days post-tMCAo, most vessels around the damaged region expressed Plexin-D1 (bottom two rows). Numbers in the red boxes indicate image order from top to bottom: 1, contralateral cortex; 2, near core damaged region; 3, peri-infarct region in the ipsilateral cortex; 4, near damaged core in the ipsilateral area. The light-colored area lacking vessels followed by AP-Sema3E binding was defined as the ischemic core (see also Supplemental Fig. 2). Scale bar = 100 μm. **e** Quantification from (**d**). (three brain sections per mouse, *n* = 5 mice/group). Data are shown as mean ± SEM. **p < 0.01, ****p < 0.0001; one-way ANOVA with Tukey’s multiple comparisons

We investigated whether Sema3E and Plexin-D1 expression are induced in ischemic tissue after tMCAo. We found a significant and rapid elevation of *Sema3e* mRNA in the peri-infarct region as early as 6 h post-tMCAo. In contrast, *Plxnd1* mRNA expression remained relatively unchanged until 24 h after ischemic injury (Fig. 1a, b). In addition, increased colocalization of *Sema3e* and the neuronal marker, *snap25*, indicated that *Sema3e* expression in peri-infarct neurons is highly responsive to ischemic injury (Fig. 1c).

High Plexin- D1 expression is found in the brain vasculature during development [43]; however, changes in its expression throughout life are not known. To examine Plexin-D1 expression in the brain vasculature, we performed an AP-Sema3E binding assay, which detects Plexin-D1 localization through specific ligand-receptor binding capacity [13, 44]. Consistent with the previous observations, Plexin-D1 was highly expressed throughout the brain vasculature. Interestingly, its expression was dramatically downregulated after birth and barely detectable in adult brain vessels, with the exception of trace amounts in a few larger vessels (Supplemental Fig. 1). Despite no change in *Plxnd1* mRNA expression during the initial phase after ischemic insult, we wondered whether Plexin-D1 could reappear in newly sprouting vessels under ischemic conditions. Since hypoxia induces VEGF and subsequent signaling via VEGFR2 upregulates *Plxnd1* expression in capillary vessels [37], we surmised that the activation of *Plxnd1* expression in remodeling vessels might take time to develop. To test this hypothesis, we analyzed the temporal features of Plexin-D1 expression after tMCAo (Fig. 1d, e). Three days after tMCAo, Plexin-D1 protein was expressed in blood vessels near the infarct core (Fig. 1d, area 2), which reached peri-infarct vessels by day 7 (Fig. 1d, area 3, and Supplemental Fig. 2). Seven days post-ischemia, most ipsilateral vessels strongly and broadly expressed Plexin-D1 (Fig. 1d, areas 3 and 4) at levels similar to those found in the early developing vasculature (Supplemental Fig. 1). This indicated that vessels were actively being reconstructed. In the contralateral (intact) hemisphere, Plexin-D1 was detected in axonal tracts (Fig. 1d, area 1) and not blood vessels.

**Fig. 2:**
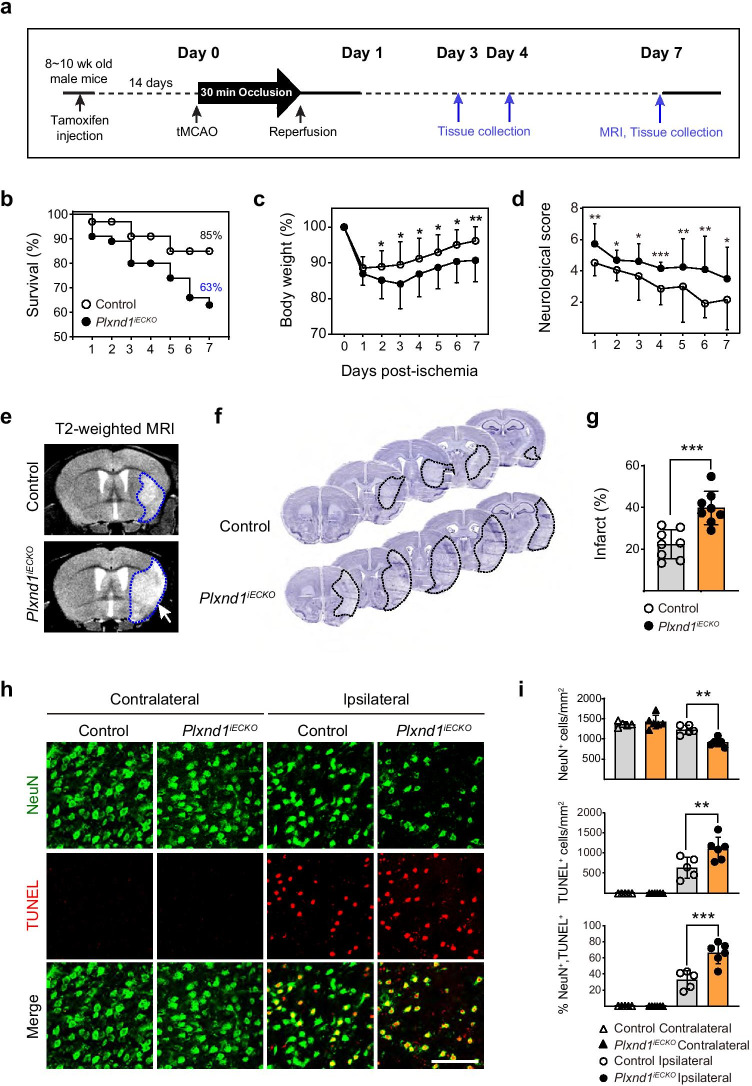
Brain endothelial cell–specific *Plxnd*1 knockout leads to severe brain damage in ischemic stroke mice **a** Schematic illustration of the experimental strategy. **b** Survival rate changes for 7 days post-tMCAo in control (Cre positive Plxnd1^*flox/*+^*)* and *Plxnd1*^*iECKO*^ mice are indicated as open and closed circles, respectively (n = 34 mice/group). **c** Body weight changes (*n* = 20 mice/group). **d** Neurological score changes. Detailed score scales are described in the “Materials and Methods“ section (*n* = 20 mice/group). **e** Representative image of T2-weighted MRI coronal sections in control and *Plxnd1*^*iECKO*^ mice. The dotted line in blue indicates the damaged area. White arrows indicate the bigger damaged brain areas in *Plxnd1*^*iECKO*^ mice. **f** Representative image galleries showing Nissl-stained brain sections from control and *Plxnd1*^*iECKO*^ mice at post-tMCAo day 7. The dotted lines in black indicate the infarction region. **g** Quantification of infarction area analysis in (**f**) (*n* = 8 mice/group). **h** Representative images of dead neurons in the peri-infarct region at post-tMCAo day 7 immunostained by NeuN (green) and TUNEL (red). In the ipsilateral area, the *Plxnd1*^*iECKO*^ mice show more TUNEL-positive cells compared to the controls. No dead cells were observed in the contralateral side. **i** Quantification from (**h**) (*n* = 5 mice/control group, *n* = 6 mice/*Plxnd1*^iECKO^ group). Data are shown as mean ± SEM. *p < 0.05, **p < 0.01, ***p < 0.001; two-tailed Student’s *t*-test (graphs **b**, **c**, **d**, and **g**), one-way ANOVA with Tukey’s multiple comparison (graph i)

### *Plxnd1* Conditional Ablation in Brain Endothelial Cells Interfered with Recovery after Ischemic Stroke

Next, to address whether Plexin-D1 was critical for vascular remodeling, we generated an endothelial brain cell–specific *Plxnd1* knockout mouse model (*Plxnd1*^*iECKO*^). Endothelial cell–specific Plexin-D1 deletion leads to significant perinatal lethality [45]; therefore, it was necessary to ablate *Plxnd1* in a temporally controlled manner. To generate this time-specific *Plxnd1* KO mouse, we crossed *Plxnd1*^*flox/flox*^ mice with *Mfsd2a-CreERT* mice, which express the tamoxifen-inducible *Cre* predominantly in brain endothelial cells and not peripheral vessels [39]. We observed that a single tamoxifen injection into the *Mfsd2a-CreERT;ROSA26-RFP* mouse sufficiently induced reporter gene expression in most adult brain endothelial cells (Supplemental Fig. 3a). Additionally, we confirmed the elimination of Plexin-D1 expression following tamoxifen injection in brain endothelial cells under hypoxic conditions using AP-Sema3E binding analysis (Supplemental Fig. 3b).

**Fig. 3:**
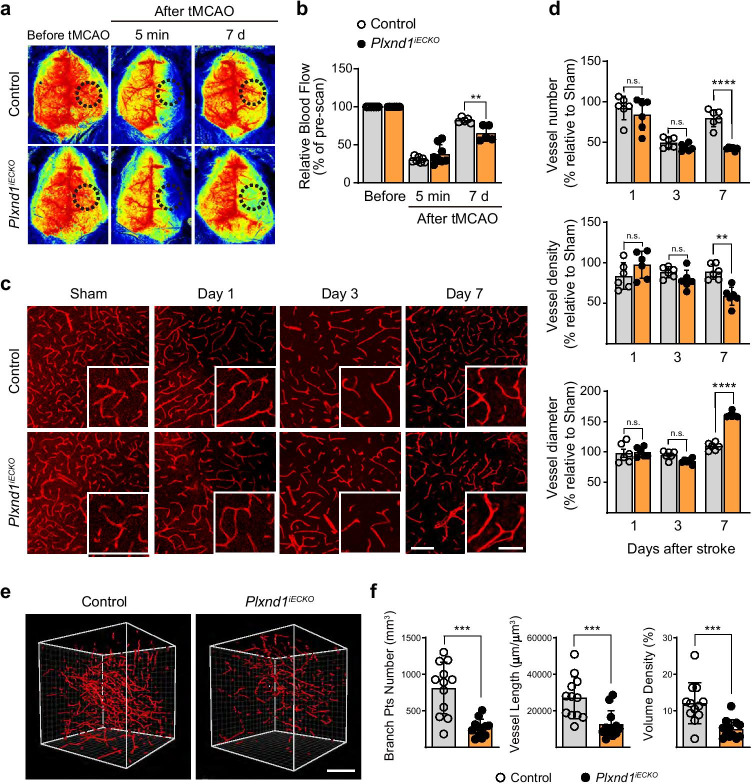
*Plxnd*1 ablation inhibits blood flow recovery and proper vascular network formation **a** Representative laser speckle images of cerebral blood perfusion in control (Cre positive Plxnd1^*flox/*+^*)* and *Plxnd1*^*iECKO*^ mouse brains. All images show areas of high and low blood perfusion as yellow–red and blue-black, respectively. The three images represent pre-tMCAo (baseline), post-tMCAo (occlusion), and post-tMCAo day 7 (after reperfusion). **b** CBF in the cerebral cortical area in (**a**) was calculated from the circular ROI (black dotted line) and quantified (*n* = 5 mice/group). **c** Representative images of temporal changes of microvessel structure in the peri-infarct region of cortex post-tMCAo from control (top panels) and *Plxnd1*^*iECKO*^ (bottom panels) mice. Inset images of each panel showed high-resolution images. Scale bars = 100 μm and 50 μm (in inset images). **d** Quantification of the microvascular number, density, and diameter for each group (*n* = 6 mice/group). Open and closed circles in black indicate control and *Plxnd1*^*iECKO*^ mice, respectively. All parameters were analyzed as a relative percentage to sham control data. **e** Representative 3D vessel reconstruction analysis images after CD31 immunostaining and tissue clearing. The whole-brain vascular network of the peri-infarct region on day 7 post-tMCAo is analyzed from control and *Plxnd1*^*iECKO*^ mouse brains in 42 × 10^6^ μm^3^ volume. Scale bars = 100 μm. **f** Quantification of branch point number, vessel length, and volume density of 3D images in (**e**) (three areas per mouse, *n* = 4 mice/group). Data are shown as mean ± SEM. **p < 0.01, ***p < 0.001, ****p < 0.0001; two-way ANOVA with Tukey’s multiple comparison (graphs in **b**, **d**) and two-tailed Student’s *t*-test (graph **f**)

We examined the extent of brain damage and vascular alterations after tMCAo in *Plxnd1*^*iECKO*^ mice following the experimental scheme outlined in Fig. 2a. Heterozygous *Mfsd2a-CreERT;Plxnd1*^*flox/*+^ littermates were used as controls for all experiments because no difference was found in blood flow, vascular structure, survival rate, or infarction size after tMCAo, just as in *Plxnd1*^*flox/flox*^ mice (Supplemental Fig. 4). In addition, we decided to perform all analyses within 7 days after stroke due to ethical considerations. First, *Plxnd1*^*iECKO*^ mice showed a significant decrease in survival rate and body weight in contrast to control littermates (Fig. 2b, c). To examine behavioral performance, we used an 8-scale neurological deficit grading system [41] and found that *Plxnd1*^*iECKO*^ mice consistently displayed a higher score than the control group for a week (Fig. 2d). On the first day post-stroke, the mean score for control mice was 4, which improved to 1–3 by post-stroke day 7. However, the mean score for *Plxnd1*^*iECKO*^ mice was 6 on day 1 after tMCAo. A relatively high neuronal deficit phenotype, i.e., spontaneous unilateral circling (scores of 4–5), was maintained a week after tMCAo.

**Fig. 4:**
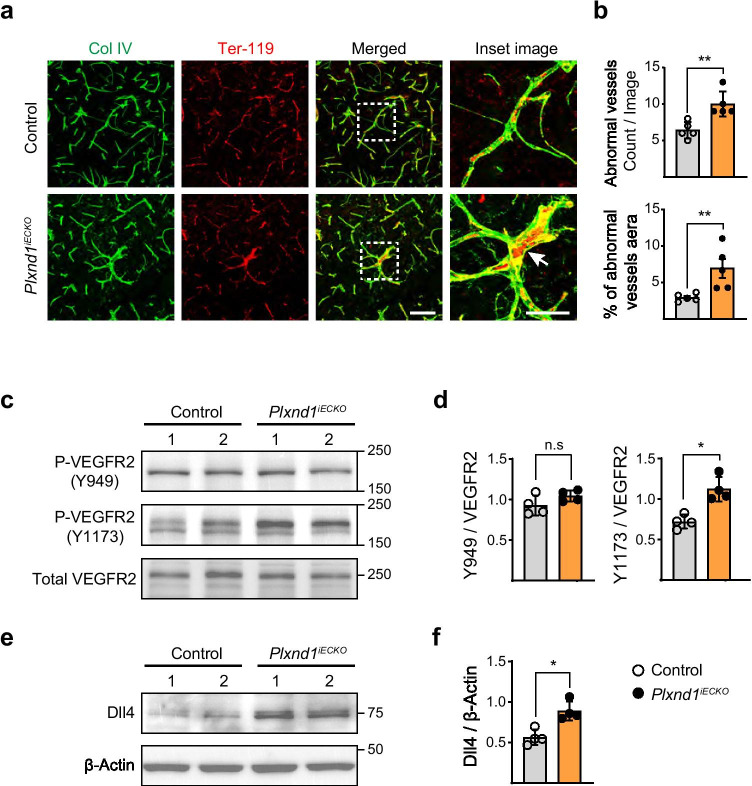
Lack of *Plxnd*1 in brain endothelial cells increases VEGF signaling leading to abnormal vascular morphology **a** Representative images of microvessel structure in the peri-infarct region at post-tMCAo day 7 from control (Cre positive Plxnd1^*flox/*+^, top panels) and *Plxnd1*^*iECKO*^ (bottom panels) mice. During recovery after stroke induction, the control mice showed typical capillary vessel structure (green, anti-Col IV) with single red blood cell inner lining (red, anti-Ter-119), whereas the *Plxnd1*^*iECKO*^ mice generated unusually thick vessels with accumulated RBCs (white arrow in bottom inset image). Boxed areas in merged panels are shown in high-resolution images (inset). Scale bars = 100 μm; high-resolution images = 50 µm. **b** Quantification of abnormal vessels from (**a**) (*n* = 5 mice/group). Open and closed circles in black indicate control and *Plxnd1*^*iECKO*^ mice, respectively (graphs in **b**, **d**, **f**). **c** Phosphorylation level of Y949 and Y1173 sites including total VEGFR2 expression analyzed by western blot at post-tMCAo day 4. Representative blots show separate peri-infarct samples from different animals per each genotype. **d** Quantification of phospho-tyrosine levels normalized by VEGFR2 expression in (**c**) (*n* = 4 mice/group). **e** Western blot images of Dll4 expression using the same samples in (**c**). **f** Quantification of Dll4 level normalized by β-actin expression (*n* = 4 mice/group). Data are shown as mean ± SEM. *p < 0.05, **p < 0.01; two-tailed Student’s *t*-test

To analyze the histological effects of tMCAo, we measured ischemia-induced lesion volume by T2-weighted MRI. We observed significantly increased high-intensity regions representing lesions in *Plxnd1*^*iECKO*^ mice on day 7 after tMCAo (Fig. 2e). In addition, the *Plxnd1*^*iECKO*^ mice showed broader infarction areas from the cortex to the striatum (Fig. 2f, g). Next, to assess the number of neurons damaged by the ischemic insult, we performed NeuN and TUNEL co-staining and found increased neuronal death in the ipsilateral, damaged hemisphere of *Plxnd1*^*iECKO*^ mice when compared with control (heterozygous *Mfsd2a-CreERT;Plxnd1*^*flox/*+^) mice. Damaged neurons were barely detectable in the contralateral side of both the control and *Plxnd1* knockout mice (Fig. 2h, i). At post-tMCAo day 7, the *Plxnd1*^*iECKO*^ mice generally showed significantly decreased neuron numbers (Fig. 2i).

### Altered Structural and Functional Cerebrovascular Remodeling in ^*Plxnd1 iECKO*^Mice after Stroke

To investigate whether reactivation of Sema3E/Plexin-D1 signaling following stroke leads to vascular alterations, we first monitored CBF in the ischemic region using laser Doppler imaging (Fig. 3a). No significant difference was observed in CBF between control and *Plxnd1*^*iECKO*^ groups before (baseline) or after occlusion (tMCAo) onset. In contrast, at 7 days after reperfusion, CBF recovery was delayed by ~ 20% in *Plxnd1*^*iECKO*^ mice when compared with control littermates (Fig. 3b).

In a rodent stroke model, active vessel sprouting begins 3 days after ischemic stroke, and noticeable angiogenesis is observed 1 week later [14, 46]. Therefore, we analyzed structural changes in the peri-infarct vasculature up to 7 days post-tMCAo. Ischemia significantly decreased the vessel number by day 3 in both heterozygous control and *Plxnd1*^*iECKO*^ mice; however, vessel number recovery was only measured in the control mice at post-tMCAo day 7 (Fig. 3c, d). *Plxnd1*^*iECKO*^ mice showed a significant decrease in vessel density at day 7 compared with control mice. Interestingly, we observed that *Plxnd1*^*iECKO*^ mice had increased overall vessel diameter and a few aberrantly thick vessels despite reduced vascular formation (Fig. 3c, d). These results suggested that Plexin-D1 expression in damaged vessels 3 days after tMCAo (Fig. 1d) is crucial for angiogenic sprouting and subsequent vascular network repair. For further in-depth analysis of the vasculature defect, we performed three-dimensional (3D) image analysis using the CUBIC method, a recently developed tissue-clearing technique [42] that allows visualization of the entire vascular network surrounding the infarct core (Fig. 3e). Overall, 3D vascular patterning was much less elaborate in the *Plxnd1*^*iECKO*^ mice; all parameters were significantly lower than those of the control mice (Fig. 3f). This emphasized that brain endothelial Plexin-D1 promotes vascular network formation following ischemic stroke.

### Aberrant VEGF Signaling Impairs Vascular Remodeling in ^*Plxnd1 iECKO*^ Mice after Stroke

*Plxnd1*^*iECKO*^ mice displayed malformed blood vessels with microaneurysm-like abnormal structures (Fig. 4a, b), suggesting a misregulation of endothelial cell proliferation and/or migration during vascular remodeling. VEGF/VEGFR2 signaling is a master regulatory pathway for vascular development and function in health and disease [47]; therefore, we assumed that Sema3E-Plexin-D1 controlled VEGF signaling after stroke. First, we examined VEGF signaling activity by measuring phosphorylated VEGFR2 expression 4 days post-tMCAo, when angiogenic remodeling actively progresses. We focused on two major tyrosine phosphorylation sites, Y949 and Y1173, that are associated with endothelial cell proliferation and migration [48]. Surprisingly, no change in the expression of phosphos-Y949 was found in the *Plxnd1*^*iECKO*^ mouse brain. In contrast, phosphorylation at Y1173 was significantly increased when compared with the control mice (Fig. 4c, d). To further assess the increased VEGF signaling, we examined Dll4 expression, a well-characterized VEGF downstream target in endothelial cells [49]. Dll4 expression was upregulated in *Plxnd1*^*iECKO*^ mice (Fig. 4e, f), which demonstrated that the absence of Plexin-D1 causes aberrant activation of VEGF signaling in vessel remodeling post-ischemia.

### Endothelial-Specific Plxnd1 Deletion Leads to BBB Breakdown

**Fig. 5:**
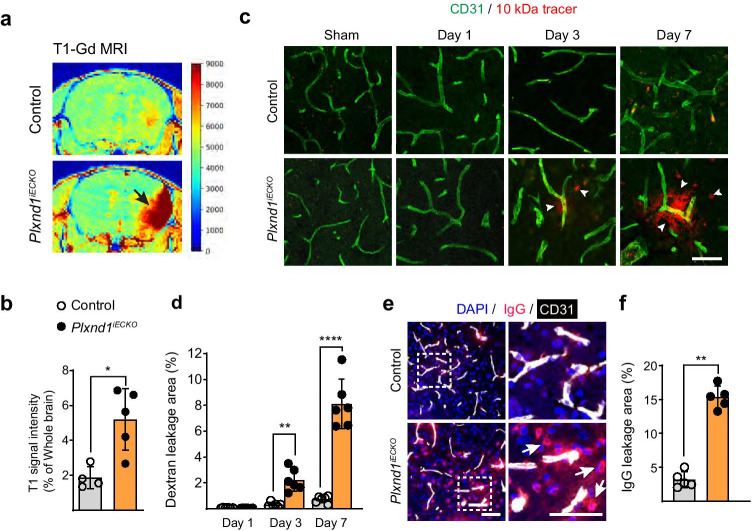
BBB breakdown during ischemia-induced vessel remodeling in *Plxnd*1 knockout mice. **a** Representative T1-Gd MRI image. Intensity of signal change between control (Cre positive Plxnd1^*flox/*+^*)* and *Plxnd1*^*iECKO*^ mouse brain parenchyma (jet image colormap). Black arrows indicate penetrable Gd-DOTA distribution in the *Plxnd1*^*iECKO*^ mice. **b** Quantitative analysis showing that Gd-DOTA enhancement was increased in the brain parenchyma of *Plxnd1*^*iECKO*^ mice, indicating increased BBB permeability (*n* = 4 mice/control group, *n* = 5 mice/*Plxnd1*^*iECKO*^ group). Open and closed circles in black indicate control and *Plxnd1*^*iECKO*^ mice, respectively (graphs in **b**, **d**, **f**). **c** Confocal microscopy imaging of 10-kDa dextran tracer (red) around CD31-positive capillaries (green) in control (top panels) and *Plxnd1*^*iECKO*^ (bottom panels) mice. Considerable tracer leakage into the brain parenchyma of *Plxnd1*^*iECKO*^ mice appeared as early as post-tMCAo day 3, whereas permeability in the control mice was barely detectable. Arrowheads indicate accumulated tracer in perivascular tissues. Scale bar = 50 μm. **d** Quantification of vascular permeability (*n* = 6 mice/group). Tracer accumulated areas were analyzed as the relative percentage of sham control (Cre positive Plxnd1^*flox/*+^ without stroke induction) values. **e** Immunostaining of IgG (red) and CD31 (white) at post-tMCAo day 7. Dotted boxes in the left panels are shown in high-resolution images on the right panels. The white arrows indicate accumulated leak IgG in the brain parenchyma of *Plxnd1*^*iECKO*^ mice. Scale bar = 100 μm. **f** Quantification of IgG-stained areas (*n* = 5 mice/group). Data are shown as mean ± SEM. *p < 0.05, **p < 0.01, ****p < 0.0001; two-tailed Student’s *t*-test (graphs in **b** and **f**) and two-way ANOVA with Tukey’s multiple comparison (graph **d**)

We examined whether the abnormal vessel formation observed in *Plxnd1*^*iECKO*^ mice during ischemic remodeling was accompanied by other functional impairments, such as BBB breakdown. To assess the integrity of growing vessels during remodeling, we assessed BBB breakdown via T1-Gd MRI in live animals. Signal intensities increased in *Plxnd1*^*iECKO*^ mice when compared with control littermates (Fig. 5a, b), indicating incomplete BBB recovery in the absence of Plexin-D1. Next, we intravenously administered a low molecular weight 10-kDa fluorescent dextran tracer and monitored its distribution within the injured cortex until post-tMCAo day 7. Tracer extravasation was observed on day 3, which continuously increased until day 7 in *Plxnd1*^*iECKO*^ mice. In control mice, 10-kDa dye leakage was barely detectable even 7 days post-tMCAo (Fig. 5c, d). In addition, we evaluated whether the impaired vessels in *Plxnd1* knockout mice were permeable to high molecular weight proteins, such as immunoglobulin G, and found a significant accumulation in the brain parenchyma (Fig. 5e, f).

**Fig. 6:**
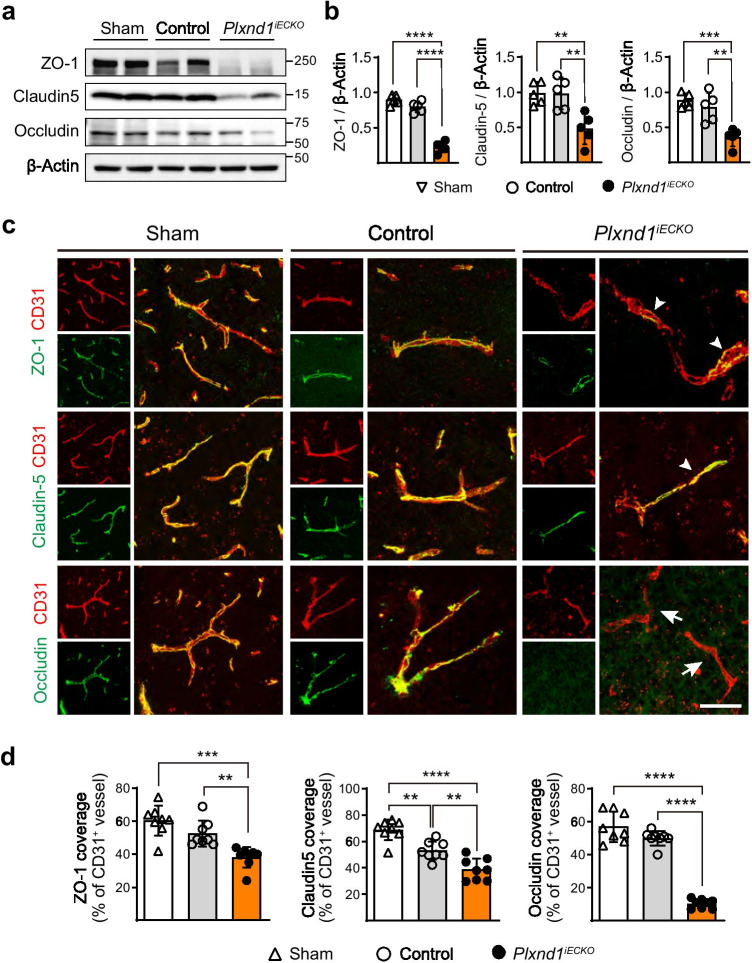
BBB breakdown in *Plxnd1* knockout mice is accompanied by reduced junctional protein expression and distribution **a** Representative western blot images of ZO-1, Claudin5, and Occludin in peri-infarct regions on post-tMCAo day 7. **b** Quantification of three junctional protein expressions shown in (**a**). Each expression level is normalized to β-Actin (*n* = 5 mice/group). Open triangles, open circles, and closed circles in black indicate sham-operated, control, and *Plxnd1*^*iECKO*^ mice, respectively (graphs in **b**, **d**). **c** Representative images of ZO-1 (green) and CD31 (red); Claudin5 (green), and CD31 (red); and Occludin (green) and CD31 (red) staining of the peri-infarct region on post-tMCAo day 7. Merged results are shown in larger images. In the *Plxnd1*^*iECKO*^ mice, some ZO-1 and Claudin-5 proteins are distributed on vessels (white arrowheads) whereas Occludin localization on vessels is barely detected (white arrows) compared with sham (Cre positive Plxnd1^*flox/*+^ without stroke induction) or control mice. Scale bar in larger images = 50 μm. **d** Quantification showing ZO-1, Claudin5, and Occludin coverage in the peri-infarct region *(n* = 8 mice/group). Data are shown as mean ± SEM. **p < 0.01, ***p < 0.001, ****p < 0.0001; one-way ANOVA with Tukey’s multiple comparison

To further investigate whether BBB disruption was caused by a structural change of the NVU, the expression of three major BBB junctional proteins was analyzed in the peri-infarct region. The expression of junctional proteins, ZO-1, Claudin5, and Occludin, was downregulated in *Plxnd1*^*iECKO*^ mice when compared with sham or control mice (Fig. 6a, b). Despite their overall downregulation in knockout mice, ZO-1 and Claudin5 were detected on remodeling vessels albeit with an altered distribution (Fig. 6c, arrowheads). The coverage ratio of Occludin on vessels lacking Plexin-D1 was extremely low (Fig. 6c arrows, d). These results demonstrated that Plexin-D1 signaling is critical to establish proper brain vascular structures because it controls the expression and distribution of BBB constituents in growing vessels.

### Inhibition of VEGF Signaling during Vascular Remodeling Facilitates Functional Recovery in ^*Plxnd1 iECKO*^ Mice

**Fig. 7:**
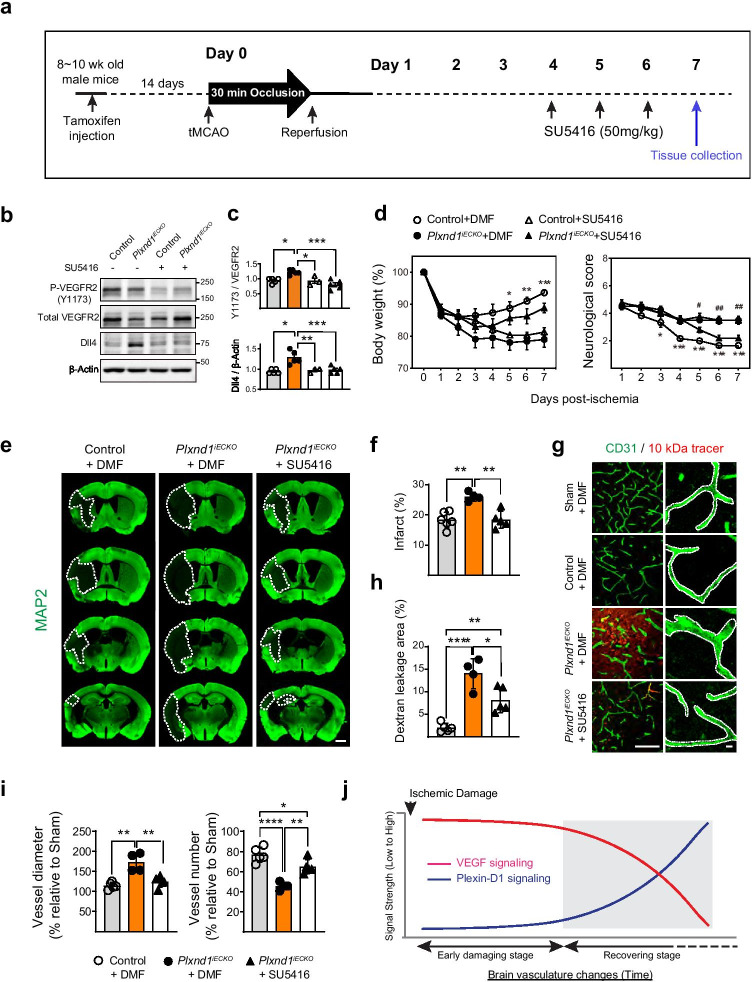
Inhibition of VEGF signaling enhances functional recovery and vascular remodeling in *Plxnd1* knockout mice **a** Schematic illustration of the experimental strategy for (**b**) to (**i**).** b** Representative western blots showing that injection of SU5416 rescued the aberrant expression of phosphorylated VEGF-R2 (Y1173) and Dll4 in the peri-infarct cortex of *Plxnd1*^*iECKO*^ mice on post-tMCAo day 7.** c** Quantification from (**b**). Each symbol marks the following (graphs in **c**, **d**, **f**, **h**, **i**): dimethylformamide (DMF)-administered control (open circles) and *Plxnd1*^*iECKO*^ mice (closed circles); VEGFR2 inhibitor, SU5416 administered-control (open triangles), and *Plxnd1*^*iECKO*^ (closed triangles) mice. **d** Body weight and neurological scores were monitored for 7 days after tMCAo (*n* = 4–6 mice/group). *p < 0.05, **p < 0.001, ***p < 0.001; control versus *Plxnd1*^*iECKO*^ + DMF groups, #*p* < 0.05, ##*p* < 0.01; *Plxnd1*^*iECKO*^ + DMF versus *Plxnd1*^*iECKO*^ + SU5416 groups. **e** Representative images of an infarction area. Brain sections were collected at post-tMCAO day 7, and the damaged brain area was analyzed by MAP2 immunostaining. Dotted lines indicate the infarction region. Scale bar = 1 mm. **f** Quantification of infraction area. **g** Representative images of vascular permeability assay (left panels) after 10-kDa dextran tracer (red) injection. In *Plxnd1*^*iECKO*^ mice, vehicle injection shows significant tracer leakage into the brain parenchyma, whereas SU5416 administration decreases tracer permeability. The dye leakage is barely detectable in the sham or control mice. Magnified images (right panels) show the CD31-positive vascular structure (green). The dotted lines demarcate vessel shape. Scale bar = 100 μm. **h** Quantification of dye leakage. The leakage area analyzed the relative percentage to sham control (Cre positive Plxnd1^*flox/*+^ without stroke induction) data. **i** Quantification of vessel diameter and number changes in (**g**) (*n* = 5 mice/control + DMF and *Plxnd1*^*iECKO*^ + SU5416 groups, *n* = 4 mice/*Plxnd1*^*iECKO*^ + DMF group). Data are shown as mean ± SEM. *p < 0.05, **p < 0.001, ****p < 0.0001; one-way ANOVA with Tukey’s multiple comparison. **j** Schematic drawing describing the relative changes of VEGF and Plexin-D1 signaling in our stroke model

Based on the above results, we speculated that reactivation of Plexin-D1 may control VEGF signaling in remodeling vessels, thereby preventing the detrimental effects of overactivated VEGF. To test this, we administered SU5416, a VEGFR2 inhibitor, during the vascular remodeling period after stroke (Fig. 7a) and analyzed VEGFR2 activation and its downstream target Dll4 expression. SU5416 injection dramatically reduced phosphor-Y1173 and Dll4 expression in *Plxnd1*^*iECKO*^ mice (Fig. 7b, c). These results provided additional evidence that signaling through VEGF2 activation in the remodeling vessel is closely modulated by the Plexin-D1 signaling pathway at the molecular level. Next, we monitored body weight and neurological score. As shown in Fig. 7d, the VEGFR2 inhibitor treatment prevented body weight loss and improved behavioral performance in *Plxnd1*^*iECKO*^ mice when compared with vehicle-treated *Plxnd1*^*iECKO*^ mice. This indicated that the functional recovery of *Plxnd1*^*iECKO*^ mice was inhibited by overactive VEGFR2 signaling. In *Plxnd1*^*iECKO*^ mice, VEGFR2 inhibition reduced infarction area (Fig. 7e, f) and BBB permeability (Fig. 7g, h). Moreover, SU5416 treatment reverted the abnormal vascular morphology in *Plxnd1*^*iECKO*^ mice (larger diameter and reduced vascular density) (Fig. 7g, i).

Finally, we tested whether the inactivation of VEGFR2 signaling within a specific time window was critical to stroke recovery. We administered SU5416 to wild-type mice before or after vascular remodeling began and analyzed the brain damage. The early inactivation of VEGF signaling caused severe brain damage, including increased edema and infarction area (~ 30%) even at day 4 post-tMCAO (Supplemental Fig. 5a–e). Moreover, late VEGF signaling inactivation during vascular recovery also caused brain damage; however, its severity was less mild (~ 15% infarction area, Supplemental Fig. 5f–j) than the early inactivation. These results indicated that VEGF signaling activation after ischemia is necessary for proper vascular remodeling, but its activation degree is differently regulated.

## Discussion

This study demonstrated that the reactivation of Sema3E-Plexin-D1 signaling following ischemic stroke is essential for reconstructing a healthy vasculature via the regulation of VEGF signaling during vascular remodeling. Therefore, enhancing Sema3E-Plexin-D1 signaling in angiogenic vessels during the critical period of vascular repair could become a new target for stroke treatment.

The expression of Sema3E and Plexin-D1 in the nervous and vascular systems changes dramatically after birth (Supplemental Fig. 1) [34]. In the developing brain, Plexin-D1 was broadly detected across capillary endothelial cells. At birth, however, its expression in vessels was sharply downregulated, and it was barely evident in adult brain vasculature (Fig. 1 and Supplemental Fig. 1). Our previous findings have demonstrated that unlike in the developing brain or peripheral vasculature, in the developing mouse retina, *Plxnd1* mRNA is expressed only in endothelial cells at the front of actively sprouting blood vessels from P3 to P5 and reappears in new sprouts to form deeper vascular layers at P6 [36, 37]. Those results, together with the results of the present study, strongly suggest that Plexin-D1 expression is practically absent in mature blood vessels.

Interestingly, Plexin-D1 expression is regulated by VEGF signaling, a major hypoxia-inducible pathway, during retinal vascularization [36]. Furthermore, it is highly upregulated in the endothelial cells of extraretinal vessels in the ischemic retina [37]. Therefore, we speculated that Plexin-D1 expression is essential in critical situations, such as vascular remodeling induced by ischemia, where newly sprouting vessels require vascular guidance in response to VEGF signaling [50]. Previous reports that have used the same ischemic stroke model found that VEGF expression was remarkably elevated in the cortex within 3 h post-ischemia. In contrast, VEGFR2 expression was high on day 1 and remained high until post-ischemia day 21 [14]. Consistent with this, vascular Plexin-D1 was observed in our study at post-ischemic insult day 3 and reached most of the damaged area in similar ischemic conditions in this study (Fig. 1d, e). This supports our assumption that Plexin-D1 expression is under VEGF signaling control, even in the brain vasculature.

Sema3E expression is confined to specific neuronal cells in the brain from development to adulthood [34, 51]. Previous studies have documented differential Sema3E expression and function under various ischemic conditions. For example, Sema3E and Plexin-D1 are upregulated in ischemic mouse limbs via p53. Inhibition of Sema3E function leads to a significant increase in blood flow recovery [52], indicating that Sema3E/Plexin-D1 signaling activation adversely affects neovascularization. In a murine ischemic retinopathy model, Sema3E expression in ganglionic cells is relatively constant. In contrast, reduced expression of aqueous Sema3E is observed in patients with diabetic retinopathy when compared with nondiabetic controls [37, 53, 54]. This suggests that the regulatory activity of Sema3E varies by pathological condition and species. In the present study, Sema3E expression was rapidly and precisely induced in peri-infarct area neurons during the early phase after stroke (Fig. 1a–c). Therefore, further study should examine how Sema3E expression is elevated in such a short period in the hypoxic brain.

Multiple studies have demonstrated that axon guidance molecules are associated with modulating vascular patterning under disease conditions and during development via similar signaling pathways [12, 55]. These guidance cues may be beneficial or detrimental during remodeling in the ischemic brain [56–60]. Sema3A has been studied during vascular recovery following ischemic damage. Stroke induction increases Sema3A in the cortex for up to 2 weeks and is detrimental to the damaged brain [61]. Additionally, Sema3A injection into the cerebral cortex increases vascular permeability, and the ablation of Sema3A expression reduces cerebrovascular permeability and brain damage in the tMCAO model [62]. Similarly, Sema3E may function as a remodeling modulator. In an oxygen-induced retinopathy model, enhanced Plexin-D1 or intravitreal Sema3E injection prevents abnormal vessel projections and leads to normal vascular remodeling [37]. This suggests a beneficial mechanism of Sema3E-Plexin-D1. In an ischemia-induced aged rat brain model, Zhou et al*.* have reported that the inhibition of Sema3E-Plexin-D1 signaling via a lentiviral shRNA knockdown system recovers the vascular network and improves physiological functions, such as cerebral perfusion and BBB integrity, thereby reducing brain damage [63]. However, the results from our current study, using genetically modified mouse models, demonstrated that a lack of Sema3E-Plexin-D1 signals in the ischemic brain leads to significant tissue damage and delayed recovery (Fig. 2). This strongly suggests a beneficial role for Sema3E-Plexin-D1 during stroke-induced functional recovery.

The underlying mechanism of Sema3E/Plexin-D1 signaling involvement in functional brain recovery after ischemic stroke is unclear. Our 3D-vascular network analysis and blood flow recovery experiment indicated that the greatest damage in the brains of *Plxnd1* knockout mice was due to aberrant and decreased vasculature (Fig. 3, 4). Previously, the absence of this signaling pathway was thought to unlock the normal limits on vessel growth, thereby increasing the size and branching of vascular networks. This has been observed in mouse peripheral and avian periocular vasculature; vessels devoid of Sema3E or Plexin-D1 grow densely across their boundaries [13, 64]. However, this mechanism is not observed in the retinal vasculature, where *Sema3e* or *Plxnd1* knockout results in sparse and underdeveloped network formation. This indicates that the diverse regulatory behaviors of Sema3E-Plexin-D1 signaling are dependent upon location and tissue. In our study, *Plxnd1* knockout mice had reduced vasculature during remodeling as opposed to the overgrowth observed in developing vasculature. Therefore, they recapitulated the retinal vasculature devoid of *Sema3e* or *Plxnd1* knockouts [36, 37].

In addition, *Plxnd1* knockout mice showed aberrantly thick remodeling vessels, which may have been caused by abnormal endothelial cell proliferation and migration (Fig. 4a, b). We analyzed phospho-VEGFR2 expression at Y1173, which is the site responsible for regulating endothelial cell proliferation and migration [48]. We found increased phosphorylation in the *Plxnd1* knockout mice (Fig. 4c, d). In addition, we examined Dll4 expression, which is associated with normal sprouting and branching as an intercellular VEGF signaling mediator [36]. Its expression was significantly increased in the *Plxnd1* knockout mice (Fig. 4e, f). These results are consistent with the previous finding that Sema3E-Plexin-D1 negatively controls VEGF signaling in the retina vasculature [36]. Considering the numerous phosphorylation sites on VEGFR2, it will be interesting to explore how the Y1173 site is under the control of Plexin-D1 signaling. Recently, Fukushima et al. [38]. have reported that Sema3E treatment results in VEGFR2 phosphorylation at Y1214, but not Y1175 (comparable to mouse Y1173), which induces reverse migration in cultured human endothelial cells. Moreover, this event occurs via RhoJ-mediated Plexin-D1 and VEGFR2 association. Therefore, it will be interesting to study whether this mechanism is recapitulated during cerebrovascular remodeling in our ischemic model.

During stroke, the VEGF signaling pathway shows both positive and negative effects in the ischemic brain according to their activation timing and dose across the initial-to-late chronic conditions [29]. Given the rapid VEGF and VEGFR2 expression after ischemic induction [14], VEGF signaling is necessary in the acute phase after stroke onset, probably acting for neuroprotection and angiogenic potentiation despite the high risk of a leaky BBB. Later, the newly sprouting vessels need to optimize the overactivated VEGF signaling to maintain proper angiogenic strength and neuroprotective roles via Sema3E-Plexin-D1. Supporting this assumption, we showed that VEGF signaling inhibition during the vascular remodeling phase promoted functional recovery accompanied by relatively normal vasculature in the *Plxnd1* knockout mice (Fig. 7). By contrast, VEGF signaling inhibition in the wild-type mice after stroke aggravated brain damage with a slight difference according to the inactivation time (Supplemental Fig. 5). In conclusion, VEGF signaling is immediately activated and prepares angiogenic sprouting during the early stages after ischemic damage, but its continuous overactivation during the recovery stage can lead to vascular injuries. To prevent the harmful effects of VEGF signaling, the reactivation of Sema3E-Plexin-D1 signaling is necessary as the damaged vasculature enters the remodeling stage (Fig. 7j). Plexin-D1 deficiency may cause excessively active VEGF signaling and induce functional and structural vascular malformations.

In this study, the disruption of Sema3E/Plexin-D1 signaling caused severe BBB breakdown. This was signified by increased tracer permeability and fewer junctional proteins (Figs. 5 and 6). BBB breakdown in response to ischemia occurs in two sequential steps: upregulation of transcytosis within hours, followed by paracellular pathway defects through the remodeling of tight junction proteins in the next 2–3 days [65]. Considering the timing of Plexin-D1 reemergence, we speculate that Sema3E-Plexin-D1 signaling is more relevant to BBB permeability through the paracellular pathway. Another mode of BBB disruption comes from the interaction impairment between endothelial cells and pericytes in the brain. During development, both cells have a reciprocal interaction through a specific signaling pathway. Endothelial cells secrete PDGF-BB to recruit pericytes expressing PDGFR-β. Conversely, pericytes secrete angiopoietin 1 (Ang1) to activate endothelial cells via Tie2 receptors [66]. Furthermore, pericytes cover most brain capillaries and continue producing matrix proteins in the basement membrane alongside endothelial cells. This promotes a stable BBB structure and impermeability [67]. Intriguingly, pericyte-derived Ang1 induces Occludin expression in brain capillary endothelial cells [66]. In this study, we found that Occludin distribution was exclusively reduced in the absence of Sema3E/Plexin-D1 signaling (Fig. 6c), which suggested a potential relationship between BBB disruption and abnormal pericytes in the *Plxnd1* knockout mice. Collectively, future studies should explore the involvement of Sema3E/Plexin-D1 signaling in the pericyte coverage process during vascular remodeling after stroke.

Here, we showed the antagonistic mechanisms of Sema3E-Plexin-D1 signaling as a beneficial mediator on VEGF-induced angiogenic sprouting. Further attention should focus on how these signaling pathways are precisely interconnected during vascular remodeling after stroke. Finally, further examination of the specific downstream targets of Sema3E-Plexin-D1 signaling in brain endothelial cells should be conducted to decipher the molecular mechanisms of intercellular communication. This may uncover novel therapeutic strategies for ameliorating the unfavorable outcomes in brain vascular diseases, such as stroke.

## Conclusion

In summary, we show that Sema3E-Plexin-D1 signaling is necessary for the development of functional brain vasculature during vascular remodeling after an ischemic insult. We found that after ischemia, neurons in the peri-infarct region upregulate Sema3E, and vessels close to the damaged region subsequently express Plexin-D1. Furthermore, loss of Plexin-D1 function exacerbated brain damage and abnormal behavioral performance, accompanied by lower vessel density in the peri-infarct region, similar to previous observations in a retinopathy model [37]. Newly generated vessels lacking Plexin-D1 also yielded abnormal BBB function and structure. Suppression of overactive VEGF signaling in the *Plxnd1* knockout during vascular remodeling periods rescued vascular defects. These data support a beneficial role of Sema3E-Plexin-D1 signaling in vascular repair regulation following ischemic stroke.

## Supplementary Information

Below is the link to the electronic supplementary material.

Figure S1 Vascular Plexin-D1 expression is downregulated after birth. Plexin-D1 protein expression in blood vessels is revealed by AP-Sema3E binding and subsequent immunohistochemistry with anti-collagen IV (Col IV). Plexin-D1 is highly expressed in all developing blood vessels at E15.5 and then dramatically downregulated at postnatal stages (white arrowheads). Red arrows in the middle panels indicate Plexin-D1-positive axonal tracks. In the adult brain, Plexin-D1 expression is silenced in all but a few larger vessels (white arrows in bottom panels). Scale bars = 200 μm (PDF 318 KB)

Figure S2 Representative image of AP-Sema3E binding analysis to delineate the ischemic core. AP-Sema3E binding images in brain sections on day 7 post-tMCAo, showing the damaged area (black dotted line) and ischemic core (red dotted line) on the ipsilateral side of the cortex. Two boxed areas are shown in the high-resolution images with a clear boundary between ischemic core and damaged tissue. Normal brain tissue is stained in purple by AP-Sema3E binding, whereas the infarction core region is slightly light-colored and lacks vessel-positive staining. Scale bars = 1 mm (PDF 49 KB)

Figure S3 Single tamoxifen injection inhibits Plexin-D1 expression in recovering brain endothelial cells **a** Reporter gene expression after tamoxifen injection into *Mfsd2a-CreERT;Rosa26-RFP* mice was analyzed in embryonic (top panels) and adult (bottom panels) mice. Red fluorescence protein (RFP) expression colocalized with CD31-positive capillaries in adult and embryonic brains. **b** AP-Sema3E binding analysis showing a significant drop in Plexin-D1 expression after tamoxifen injection in the *Plxnd1*^*iECKO*^ mouse. Scale bars in (a) and (b) = 100 μm (PDF 299 KB)

Figure S4 The heterozygous brain endothelial-specific *Plxnd1* knockout is indistinguishable from Cre-negative littermate mice a Blood flow analysis from Cre-negative *Plxnd1*^*flox/flox*^ (wild-type) and *Mfsd2a-CreERT; Plxnd1*^*flox/*+^(heterozygous) littermates. Under normal breeding conditions, there is no difference between wild-type and heterozygous mice (n = 5 mice/group). **b** No vascular structural differences are observed between the two groups in (a). The vessel area, length, and branch point numbers were analyzed (n = 12 mice/*Plxnd1*^*flox/flox*^ group, n = 15 mice/*Mfsd2a-CreERT; Plxnd1*^*flox/*+^). **c** Survival rate monitoring after tMCAo between the two groups. Circles and triangles indicate *Plxnd1*^*flox/flox*^ and *Mfsd2a-CreERT; Plxnd1*^*flox/*+^, respectively. There was no significant difference between the two groups (n = 6 mice/group). **d** At post-tMCAo day 7, no difference in infarction degree was found between groups, as measured by MAP2 immunostaining (n = 4 mice/group). Data are shown as mean ± SEM. n.s., non-significant; two-tailed Student’s *t*-test. Scale bars in (b) = 100 μm; (d) = 1 mm (PDF 168 KB)

Figure S5 Inhibition of VEGF signaling after ischemic damage worsened brain damage **a** Schematic illustration of the experimental strategy for (b) to (e). The VEGFR2 inhibitor, SU5416, was administered at days 1, 2, 3 after tMCAo induction, and the brain was isolated on day 4 for further analysis. **b** At day 4 post-tMCAo, the infarction region was analyzed by MAP2 immunostaining. The dotted lines indicate the infarction region. **c** SU5416 injection induces hemorrhagic vascular damage (arrowheads) when compared with the vehicle-injected controls. **d** Quantification of the infarction area (n = 4 mice/group). **e** Quantification of edema. Early inhibition of VEGF signaling causes serious brain damage (n = 4 mice/group).** f** Schematic illustration of the experimental strategy for (g) to (j). The VEGFR2 inhibitor, SU5416, was administered at days 4, 5, 6 after tMCAo induction, and the brain was isolated on day 7 for further analysis. **g** At day 7 post-tMCAo, the infarction region was analyzed by MAP2 immunostaining. The dotted lines indicate the infarction region. **h** Quantification of the infarction area (n = 4 mice/group). **i** Body weight changes. SU5416 injection significantly reduced body weight (n = 4 mice/group). **j** Neurological score changes. SU5416 injection worsened behavioral performances. Detail score scales are described in the Materials and Methods (n = 4 mice/group). Data are shown as mean ± SEM. *p < 0.05; two-tailed Student’s *t*-test. Scale bars in (b) and (g) = 1 mm; (c) = 5 mm (PDF 226 KB)

## Data Availability

All data generated and analyzed for this study are included in this published article and its supplementary additional files.

## Abbreviations

AP: Alkaline phosphatase
BBB: Blood-brain barrier
CBF: Cerebral blood flow
CCA: Common carotid artery
ICA: Internal carotid artery
ISH: In situ hybridization
MCAo: Middle cerebral artery occlusion
MRI: Magnetic resonance imaging
NVU: Neurovascular unit
PFA: Paraformaldehyde
SEM: Standard error of the mean
VEGF: Vascular endothelial growth factor
