# Supplementary figures and images for: Vascular Sema3E-Plexin-D1 Signaling Reactivation Promotes Post-stroke Recovery through VEGF Downregulation in Mice
Source: Transl Stroke Res. 2021 May 12;13(1):142–59. doi: 10.1007/s12975-021-00914-4 (PMC8766426; doi:10.1007/s12975-021-00914-4)

Fig S1.

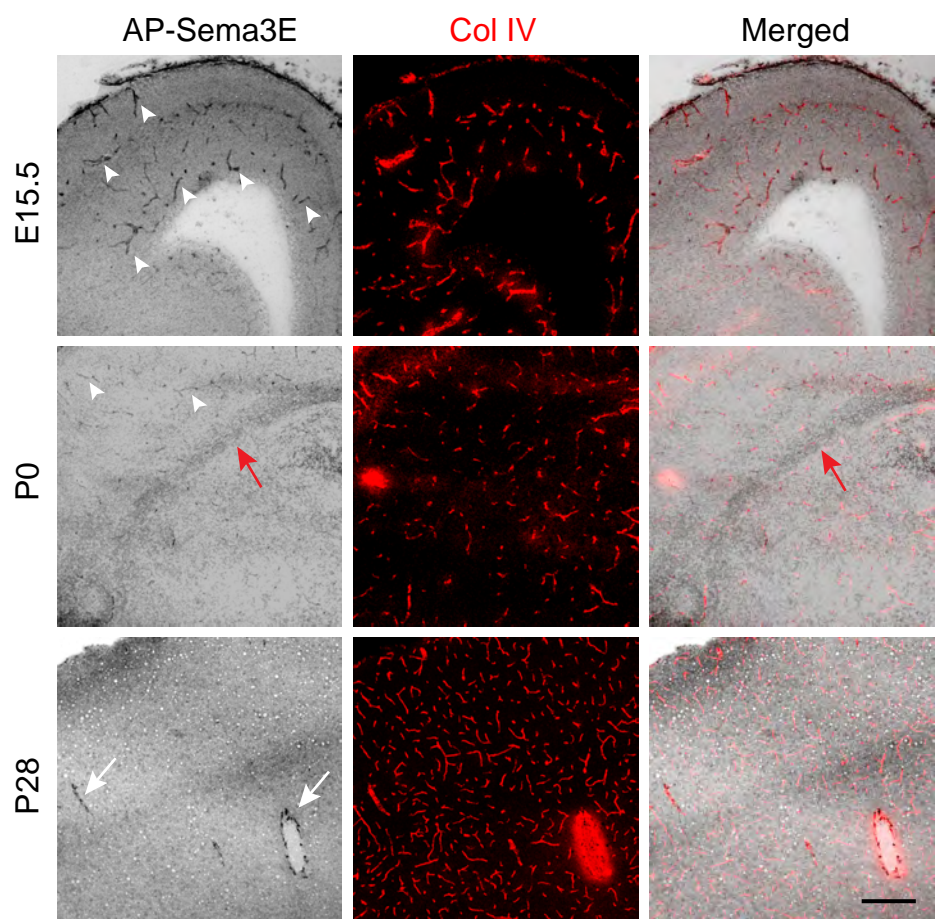

Supplement: Supplementary file 1 — Vascular Plexin-D1 expression is downregulated after birth. Plexin-D1 protein expression in blood vessels is revealed by AP-Sema3E binding and subsequent immunohistochemistry with anti-collagen IV (Col IV). Plexin-D1 is highly expressed in all developing blood vessels at E15.5 and then dramatically downregulated at postnatal stages (white arrowheads). Red arrows in the middle panels indicate Plexin-D1-positive axonal tracks. In the adult brain, Plexin-D1 expression is silenced in all but a few larger vessels (white arrows in bottom panels). Scale bars = 200 μm (PDF 318 KB) [file 12975_2021_914_MOESM1_ESM.pdf]

Fig S2.

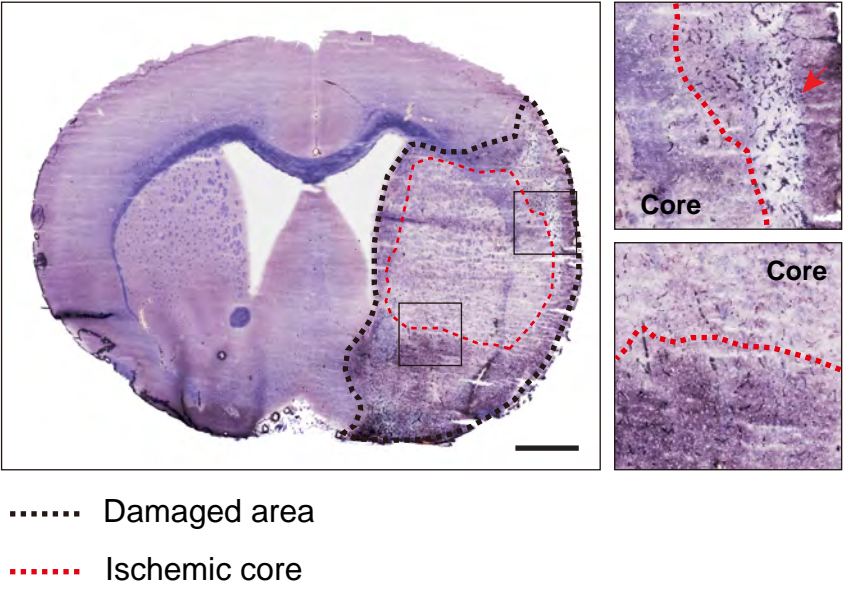

Supplement: Supplementary file 2 — Representative image of AP-Sema3E binding analysis to delineate the ischemic core. AP-Sema3E binding images in brain sections on day 7 post-tMCAo, showing the damaged area (black dotted line) and ischemic core (red dotted line) on the ipsilateral side of the cortex. Two boxed areas are shown in the high-resolution images with a clear boundary between ischemic core and damaged tissue. Normal brain tissue is stained in purple by AP-Sema3E binding, whereas the infarction core region is slightly light-colored and lacks vessel-positive staining. Scale bars = 1 mm (PDF 49 KB) [file 12975_2021_914_MOESM2_ESM.pdf]

Fig S3.

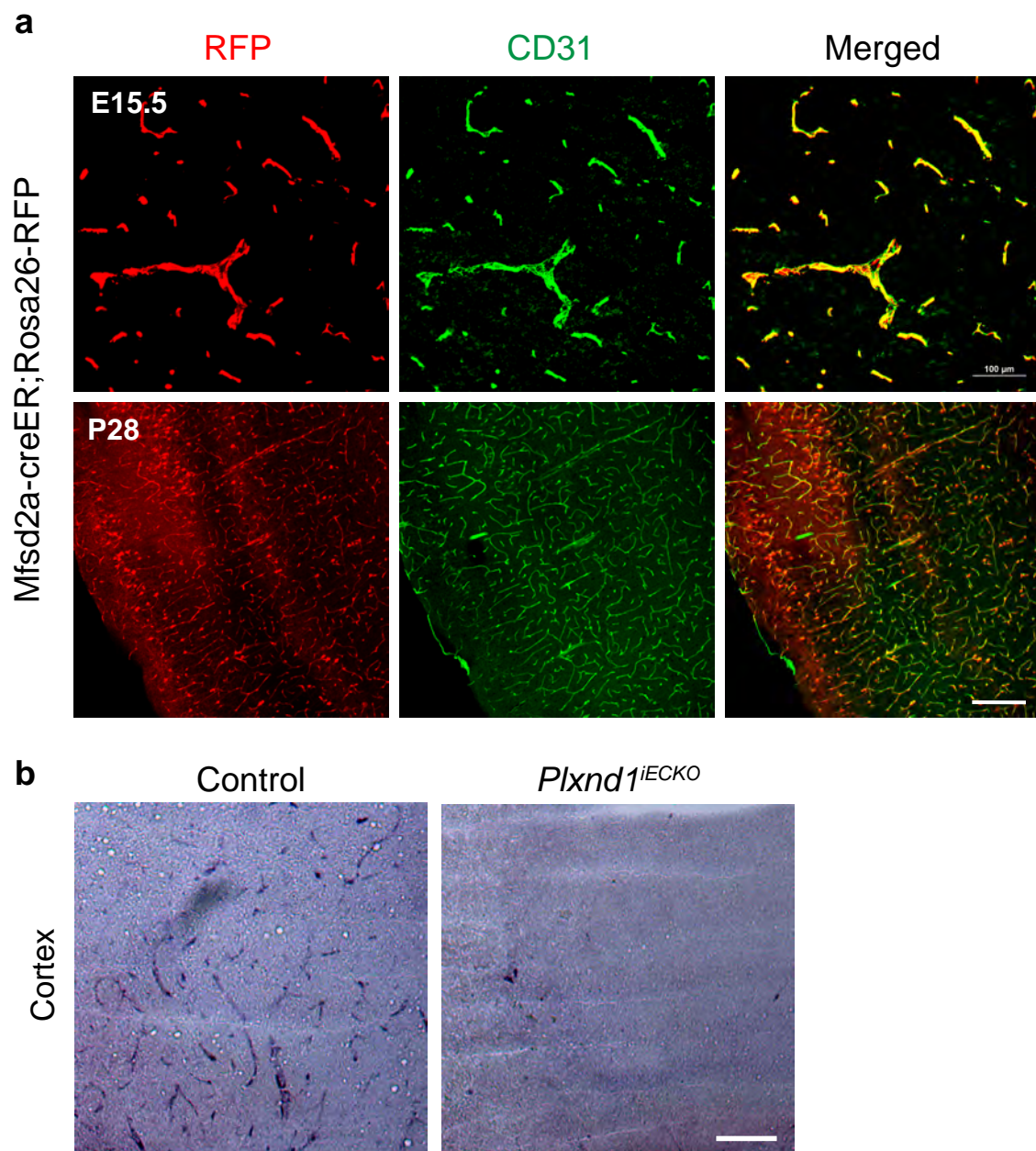

Supplement: Supplementary file 3 — Single tamoxifen injection inhibits Plexin-D1 expression in recovering brain endothelial cells a Reporter gene expression after tamoxifen injection into Mfsd2a-CreERT;Rosa26-RFP mice was analyzed in embryonic (top panels) and adult (bottom panels) mice. Red fluorescence protein (RFP) expression colocalized with CD31-positive capillaries in adult and embryonic brains. b AP-Sema3E binding analysis showing a significant drop in Plexin-D1 expression after tamoxifen injection in the Plxnd1iECKO mouse. Scale bars in (a) and (b) = 100 μm (PDF 299 KB) [file 12975_2021_914_MOESM3_ESM.pdf]

Fig S4.

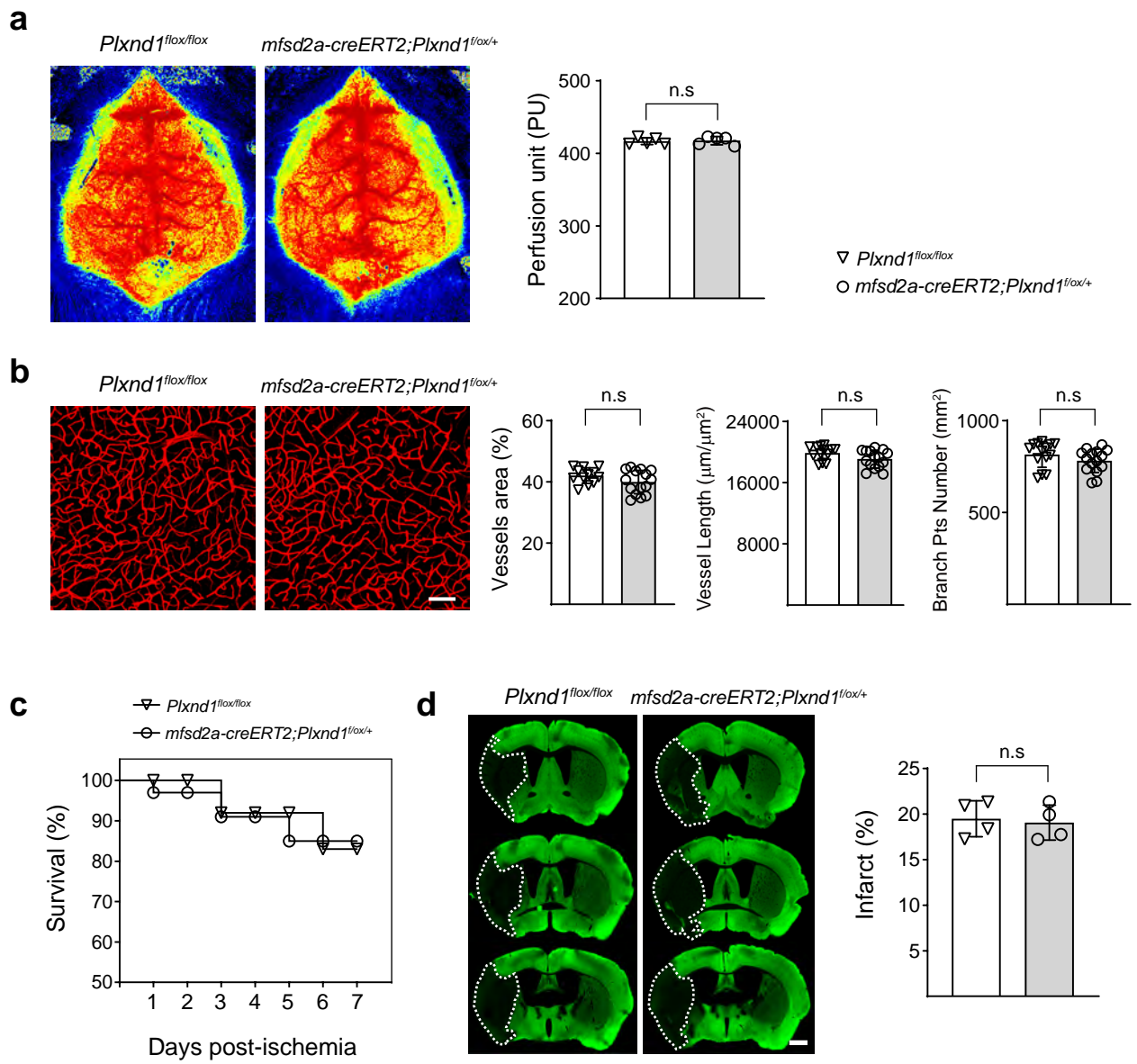

Supplement: Supplementary file 4 — The heterozygous brain endothelial-specific Plxnd1 knockout is indistinguishable from Cre-negative littermate mice a Blood flow analysis from Cre-negative Plxnd1flox/flox (wild-type) and Mfsd2a-CreERT; Plxnd1flox/+(heterozygous) littermates. Under normal breeding conditions, there is no difference between wild-type and heterozygous mice (n = 5 mice/group). b No vascular structural differences are observed between the two groups in (a). The vessel area, length, and branch point numbers were analyzed (n = 12 mice/Plxnd1flox/flox group, n = 15 mice/Mfsd2a-CreERT; Plxnd1flox/+). c Survival rate monitoring after tMCAo between the two groups. Circles and triangles indicate Plxnd1flox/flox and Mfsd2a-CreERT; Plxnd1flox/+, respectively. There was no significant difference between the two groups (n = 6 mice/group). d At post-tMCAo day 7, no difference in infarction degree was found between groups, as measured by MAP2 immunostaining (n = 4 mice/group). Data are shown as mean ± SEM. n.s., non-significant; two-tailed Student’s t-test. Scale bars in (b) = 100 μm; (d) = 1 mm (PDF 168 KB) [file 12975_2021_914_MOESM4_ESM.pdf]

Fig S5.

**a**

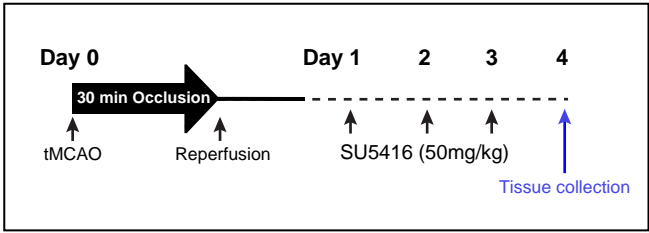

**b**

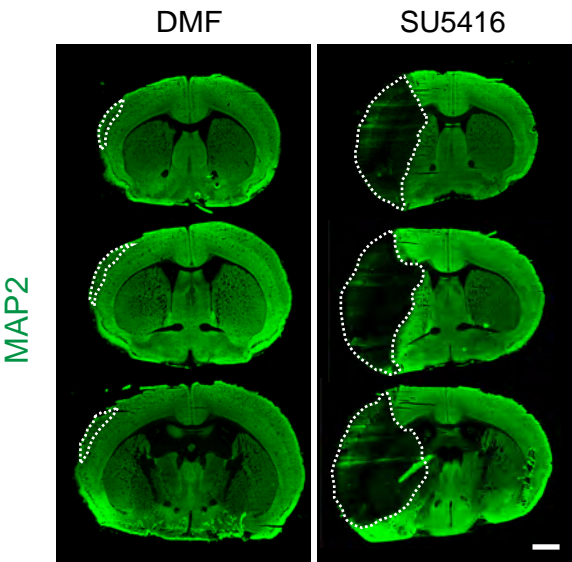

**c**

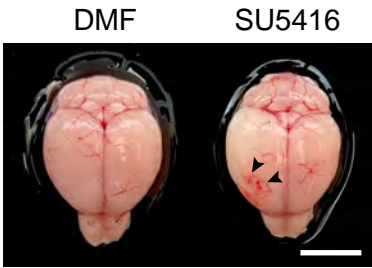

**d**

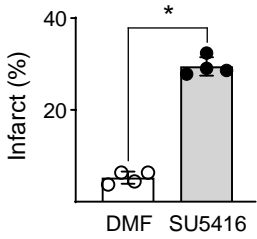

**e**

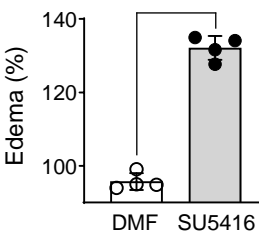

**f**

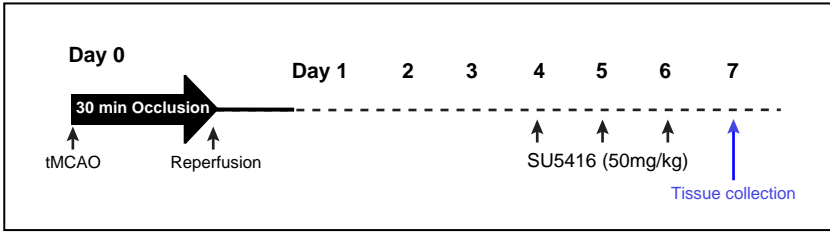

**g**

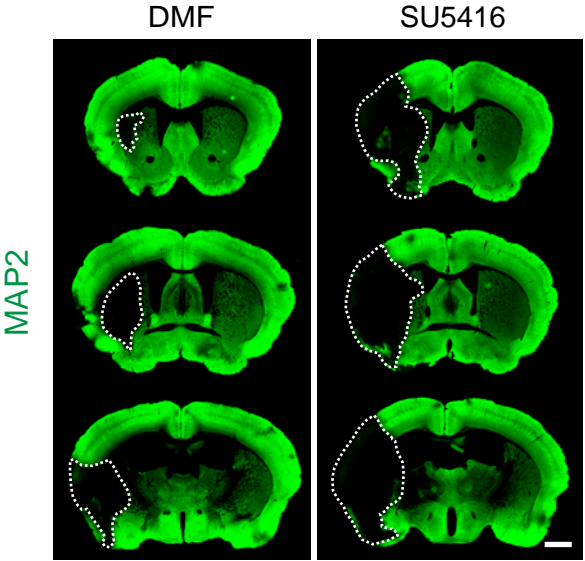

**h**

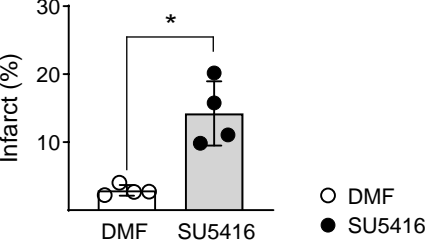

**i**

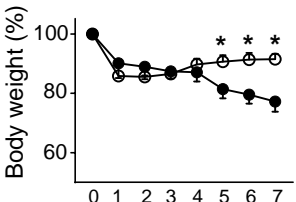

**j**

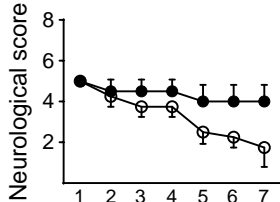

Supplement: Supplementary file 5 — Inhibition of VEGF signaling after ischemic damage worsened brain damage a Schematic illustration of the experimental strategy for (b) to (e). The VEGFR2 inhibitor, SU5416, was administered at days 1, 2, 3 after tMCAo induction, and the brain was isolated on day 4 for further analysis. b At day 4 post-tMCAo, the infarction region was analyzed by MAP2 immunostaining. The dotted lines indicate the infarction region. c SU5416 injection induces hemorrhagic vascular damage (arrowheads) when compared with the vehicle-injected controls. d Quantification of the infarction area (n = 4 mice/group). e Quantification of edema. Early inhibition of VEGF signaling causes serious brain damage (n = 4 mice/group). f Schematic illustration of the experimental strategy for (g) to (j). The VEGFR2 inhibitor, SU5416, was administered at days 4, 5, 6 after tMCAo induction, and the brain was isolated on day 7 for further analysis. g At day 7 post-tMCAo, the infarction region was analyzed by MAP2 immunostaining. The dotted lines indicate the infarction region. h Quantification of the infarction area (n = 4 mice/group). i Body weight changes. SU5416 injection significantly reduced body weight (n = 4 mice/group). j Neurological score changes. SU5416 injection worsened behavioral performances. Detail score scales are described in the Materials and Methods (n = 4 mice/group). Data are shown as mean ± SEM. *p < 0.05; two-tailed Student’s t-test. Scale bars in (b) and (g) = 1 mm; (c) = 5 mm (PDF 226 KB) [file 12975_2021_914_MOESM5_ESM.pdf]
